# Special Issue “The Role of Mesenchymal Stem Cells on Inflammatory and Fibrotic Diseases”

**DOI:** 10.3390/ijms24108578

**Published:** 2023-05-11

**Authors:** Mariangela Di Vincenzo, Monia Orciani

**Affiliations:** Department of Clinical and Molecular Sciences—Histology, Università Politecnica delle Marche, 60126 Ancona, Italy; m.divincenzo@pm.univpm.it

This Special Issue focused on the complex role played by MSCs in the onset and development of inflammatory diseases: MSCs can support or counteract inflammation and, in turn, the onset of disease. However, among the eight original articles and six reviews published here, only one original article focused on the pro-inflammatory effects of MSCs, while all others proposed MSCs as a useful tool for reducing inflammation and as a suitable candidate for cell therapy.

The works published here cover different pathologies, ranging from epilepsy to lung and heart diseases and considering different sources of MSCs, such as adipose tissue (ADSC or ASC), bone marrow (BM-MSCs) and others.

In the review published by Brave et al. [1], the authors outline the current knowledge on cell therapy with MSCs in the treatment of lung diseases (COPD, COVID-19 and ARDS). MSCs are able to reprogram the immune response and to reduce the production of pro-inflammatory cytokines, favouring the repair of lung injury and inhibiting apoptosis and pathological remodelling. The authors compare different strategies, such as the use of MSCs or their conditioned medium or secretome, as well as different routes of administration. They conclude that cell therapy with MSCs and/or their derivatives is an interesting new approach for selected pulmonary diseases, reducing overall inflammation; nevertheless, regulatory approval processes are still mandatory. On the other hand, Bonifazi et al. report that MSCs drive an inflamed microenvironment in idiopathic pulmonary fibrosis (IPF) [2]. MSCs isolated from the lung of patients with IPF (IPF-MSCs) express higher levels of genes involved in inflammation, oxidative stress and extracellular matrix formation than the MSCs of control subjects (C-MSCs). Furthermore, IPF-MSCs induce a pathological phenotype in lung fibroblast (NHLF) when co-cultured, underlying how MSCs could sustain the onset and the development of IPF. 

Other works specifically focus on ADSC and their protective/reparative effect on other cells, modulating inflammation and immunity. Rochette et al., in their review, consider the role of miRNAs inside EVs (extracellular vesicles) that, such as the canonical chemokines, may act as a new mechanism of intercellular crosstalk [3]. In addition, Rozier et al. report the anti-fibrotic effects of ASC and their EVs in an in vitro model of systemic sclerosis (SSc) [4]. The treatment of human fibroblast with TGF-β reproduces the characteristics of fibroblasts isolated from SSc patients. Co-culture with ASC and even more with related EVs reduces the fibrotic phenotype through the regulation of αSMA, COL1A1 and MMPs. The protective effects of ADSC on macrophages exposed to LPS and IFN-γ are also proven by He et al. [5]. In this work ADSC’s cell membranes are removed via ultrasonication to obtain membrane-free stem cell extract (MFSC-Ex). The pre-treatment of RAW 264.7 macrophages with MFSC-Ex inhibits inflammation and oxidative stress induced by LPS/IFN-γ-exposure through the regulation of NF-κB and MAPK signalling pathways. At the same time, a study conducted by Carceller et al. demonstrates that mouse-derived ASC secretome has anti-inflammatory effects on mouse-derived peritoneal macrophages, when stimulated with LPS, by reducing the translocation of nuclear factor-κB (NF-kB) into the nucleus [6]. They tested the conditioned medium from ASC with EV (CM) or without EV (CM-EV), and curiously, no differences were found regarding the reduction of inflammation, revealing that the anti-inflammatory effects are not related to EVs.

It appears evident that further research is needed to understand the mechanisms underlying the immunomodulatory and endocrine properties of ADSCs and to confirm their use for the treatment of pathologies characterised by chronic inflammation. With this in mind, Holthaus et al. reveal how MSCs can drive the polarization and reprogram macrophages’ profile [7]. They treated M1 and M2a differentiated murine macrophages with BM-MSCs secretome, either preconditioned (preMSC-CM) or not (MSC-CM) with pro-inflammatory factors (IFN-γ, IL1-β). Their results show that preMSC cm mostly suppresses pro-inflammatory cytokines in M1 and improves the anti-inflammatory state by driving the polarization of M2a to M2b and M2c profiles that secrete high levels of anti-inflammatory IL-10.

The anti-inflammatory properties of MSCs were reviewed also by Salary et al. and Pignattaro et al. [8,9]. Their first work offers a new point of view on the treatment of epilepsy, no longer considered to be a purely neurological disorder but rather as a pathology displaying chronic inflammation. It seems that MSCs reduce epileptogenesis through the release of trophic and anti-inflammatory factors, which partially restore the physiological conditions in the damaged areas. Authors point out that, despite all the information from in vitro or in vivo mouse models, the knowledge concerning human clinical application is still completely inadequate. Likewise, Pignattaro et al. deal with neurodegenerative disorders and recall a previous study on a rat model of Parkinson’s disease, a neuroinflammatory pathology. They demonstrate the ability of BM-MSCs to migrate in the damaged brain area, exerting a protective effect on dopaminergic neurons against apoptosis and degeneration, with a significant improvement of the symptomatology.

Another review summarises the properties of MSCs (derived from bone marrow, adipose tissue and umbilical cord) in fibrotic heart disease regarding the release of growth factors, inflammatory mediators, microRNAs and cytokines, directly or inside EVs [10]. The effects of the secreted factors involve different pathways: a reduction in inflammation as well as fibrosis by modulating MMPs, a decrease in collagen deposition and the inhibition of the conversion of cardiac fibroblasts to myofibroblasts and the stimulation of angiogenesis. Pre-clinical models confirm that MSCs treatments positively affect cardiac function. The anti-fibrotic effects related to the modulation of MMPs were also supported by Choi et al. [11]. They demonstrated that MMP-1 expression by MSCs isolated from Wharton’s jelly (WJ-MSCs) is crucial to reducing collagen deposition in a hydrogen peroxide-induced fibrosis myotube model used to study Duchenne muscular dystrophy (DMD).

The differentiation properties of MSCs are instead discussed by Kwon et al. [12]. The authors report that MSCs can be used to induce cartilage regeneration following two different approaches: the transplantation of MSCs, which can directly differentiate into chondrocytes or secrete anti-inflammatory/anti-apoptotic factors, and the use of MSCs-derived exosomes, containing miRNAs and proteins able both to promote chondrogenesis and to inhibit inflammation and apoptosis. Similarly, Willems et al. focus on the mechanisms involved into the differentiation of human cardiac stem cells isolated from atrial appendages (CASCs) [13]. Their study investigates whether the activation and the inhibition of Wnt/β-catenin signalling are crucial for the proliferation and maturation of CASCs, respectively. The results show that the stimulation of Wnt/β-catenin improves the expression of cardiac differentiation markers but does not affect proliferation, and its inhibition does not induce mature myocardial differentiation, confirming that Wnt signalling has limited effects on CASC proliferation and differentiation.

Yu et al. highlight that a dysfunctional microenvironment could impair MSCs homeostasis; specifically, after treatment with inflammatory cytokines, such as IFN-γ and TNF-α, human placenta-derived MSCs (hPD-MSCs) are overactivated, and the treatment with MIT001, a small anti-inflammatory and anti-necrotic molecule, is able to restore mitochondrial functions with a reduction in basal respiration, ATP production and cellular ROS levels [14]. The articles/reviews collected in this Special Issue describe a multifaceted role for MSCs that may act by promoting or suppressing inflammation and dysregulated immune response. It appears evident that the different microenvironments in which they mature shape their behaviour by drawing a complex picture, and more in-depth knowledge of the biology of MSCs is needed to evaluate their use in cell therapy.
